# Nitrogen and fatty acid rumen metabolism in cattle offered high or low polyphenol oxidase red clover silage

**DOI:** 10.1017/S1751731118003294

**Published:** 2018-12-19

**Authors:** M. R. F. Lee, R. Fychan, J. K. S. Tweed, N. Gordon, V. Theobald, R. Yadav, A. Marshall

**Affiliations:** Institute of Biological, Environmental and Rural Science, Aberystwyth University, Gogerddan Campus, Aberystwyth, Ceredigion SY23 2EB, UK

**Keywords:** plant enzymes, forage legumes, trifolium pratense, C18 polyunsaturated fatty acid biohydrogenation, nitrogen use efficiency

## Abstract

Polyphenol oxidase (PPO) in red clover (RC) has been shown to reduce both lipolysis and proteolysis in silo and implicated (*in vitro*) in the rumen. However, all *in vivo* comparisons have compared RC with other forages, typically with lower levels of PPO, which brings in other confounding factors as to the cause for the greater protection of dietary nitrogen (N) and C18 polyunsaturated fatty acids (PUFA) on RC silage. This study compared two RC silages which when ensiled had contrasting PPO activities (RC+ and RC−) against a control of perennial ryegrass silage (PRG) to ascertain the effect of PPO activity on dietary N digestibility and PUFA biohydrogenation. Two studies were performed the first to investigate rumen and duodenal flow with six Hereford×Friesian steers, prepared with rumen and duodenal cannulae, and the second investigating whole tract N balance using six Holstein-Friesian non-lactating dairy cows. All diets were offered at a restricted level based on animal live weight with each experiment consisting of two 3×3 Latin squares using big bale silages ensiled in 2010 and 2011, respectively. For the first experiment digesta flow at the duodenum was estimated using a dual-phase marker system with ytterbium acetate and chromium ethylenediaminetetraacetic acid as particulate and liquid phase markers, respectively. Total N intake was higher on the RC silages in both experiments and higher on RC− than RC+. Rumen ammonia-N reflected intake with ammonia-N per unit of N intake lower on RC+ than RC−. Microbial N duodenal flow was comparable across all silage diets with non-microbial N higher on RC than the PRG with no difference between RC+ and RC−, even when reported on a N intake basis. C18 PUFA biohydrogenation was lower on RC silage diets than PRG but with no difference between RC+ and RC−. The N balance trial showed a greater retention of N on RC+ over RC−; however, this response is likely related to the difference in N intake over any PPO driven protection. The lack of difference between RC silages, despite contrasting levels of PPO, may reflect a similar level of protein-bound-phenol complexing determined in each RC silage. Previously this complexing has been associated with PPOs protection mechanism; however, this study has shown that protection is not related to total PPO activity.

## Implications

Red clover (RC) silage has been shown to improve nitrogen use efficiency (NUE; dietary N retention) and C18 polyunsaturated fatty acid (PUFA) retention via lower hydrolytic loss in silage and across the rumen, with polyphenol oxidase (PPO) proposed as a mode of action. However, it was not known if increasing the activity of PPO could result in a further improvement to inform RC breeding programmes. No improvement in NUE or PUFA was found between high and low PPO RCs when offered to beef steers and non-lactating dairy cows.

## Introduction

In a review by Lee (2014), RC (*Trifolium pratense*) PPO’s protection of plant protein in the rumen was reported to be through the complexing of leaf proteins in the formation of protein-bound phenols (PBP) rather than protease deactivation *per se*. Numerous studies with RC have shown a significant reduction of rumen ammonia-N release per unit of dietary N consumed compared with other forages (Dewhurst *et al*., 2003; Merry *et al*., 2006; Vanhatalo *et al*., 2009). This indicates a lower degradability of RC protein in the rumen, which may be due to PPO induced PBP complexing reducing protein solubility.

A further benefit of RC silage is a reduction in C18 PUFA biohydrogenation compared to grass silage (Lee *et al*., 2003) with a subsequent improvement in the fatty acid composition of ruminant livestock products. Polyphenol oxidase reduces plant mediated lipolysis by inhibiting plant lipases in silo through complexing, that is, PBP formation (Van Ranst *et al*., 2011). However, reduced activity of plant lipases would have little effect on PUFA biohydrogenation in the rumen due to the greater concentration of rumen microbial lipases (Harfoot and Hazelwood, 1988). In the review by Lee (2014), the potential mechanisms for protecting PUFA were assessed as either-or combination of entrapment within PBP reducing access to microbial lipases or differences in rumen digestion kinetics of the forage. Recently, Gadeyne *et al*. (2015) showed that protein extracts rich in PPO could protect PUFA against biohydrogenation providing strong evidence for the protective role of PBP emulsions in the rumen.

The potential to increase NUE and reduce PUFA biohydrogenation in RC may therefore be realised through the breeding of elevated levels of PPO. The objective of this study was to test whether RC with higher PPO activity would increase NUE (N retention) and reduce PUFA biohydrogenation compared to a RC with lower PPO activity.

## Material and methods

### Experimental design – experiment 1 – rumen nitrogen metabolism and digestibility

A similar design was previously reported by Lee *et al*. (2014) in brief: six Hereford×Friesian steers (623±33.6 kg), prepared with a rumen cannula and a simple ‘T’-piece cannula in the proximal duodenum, were allocated to either: standard PPO RC silage (RC+); low PPO RC silage (RC−) or perennial ryegrass silage (PRG; *Lolium perenne*). The silages were offered at a daily rate of 14 to 15 g dry matter (DM)/kg BW to each animal during the measurement period. The experiment consisted of two 3×3 Latin squares with each period 21 days in duration, consisting of 14 days of adaptation to the diet, 6 days for duodenal marker infusion of which collection was on the last 2 days, and 1 day for rumen sampling.

Animals were weighed before allocation to treatment and at the end of each period with BW used to determine feed offered for each steer during the subsequent period. Silage DM was determined at each mixing of the big bales to provide silage for each period. For RC, a big bale from the first and second cut were bulked for each period (see below). Daily feed allocations were offered in two equal meals at 0800 and 1600 h. Any refusals were removed at 0745 h and sub-sampled so that actual DM intake could be determined. Animals were individually penned and had free access to fresh water and mineral blocks (Baby Red Rockies; Winsford, Cheshire, UK).

Information relating to the sowing and maintenance of the swards is reported in the Supplementary Material S1. First cut big bale silage for the RC was cut on 26 May 2010 and second cut on 10 August 2010 wilted for 48-h and ensiled with Powerstart at a rate of 2 litre/t fresh weight (FW) to supply ca. 1×10^12^ colony forming units (Genus PLC, Crewe, Cheshire, UK). Grass was cut on 25 May 2010 using a mower conditioner and then spread and baled after a 24-h wilt with no additive. Bales were wrapped with four layers of film wrap 750 mm wide 25 µm thick and stored on farm until first opening in April 2011. Samples of RC were taken as cut for PPO enzyme activity and phenol content and as baled for PBP analysis.

### Sample collection methods – experiment 1

A representative silage sample of each forage per period was collected by sub-sampling (ca. 200 g FW) daily and bulking per period which was maintained frozen at −20°C before analysis.

Digesta flow at the duodenum was estimated using a dual-phase marker system with ytterbium acetate as the particulate and chromium ethylenediaminetetraacetic acid (EDTA) as the liquid phase markers (Faichney, 1975). The procedure was as described by Lee *et al*. (2014) with the exceptions that on day 14 of each period, duodenal background samples (250 ml) were collected and a whole and a centrifuged digesta sample prepared, freeze-dried and analysed. On day 15, ytterbium acetate (576±3.3 ppm) and chromium EDTA (2 797±120.6 ppm) infusions commenced via the rumen cannula at a rate of 28.7±0.04 and 28.3±0.09 ml/h, respectively. Duodenal digesta (250 ml at each collection) was collected on days 19 and 20, manually every 3-h interval over each 24-h period, bulked across the day and a whole and centrifuged sample prepared as described above. Hourly rumen fluid (10 ml) was collected on day 21 (0800 to 1700 h) with pH and ammonia-N determined as described by Lee *et al*. (2014).

### Experimental design – experiment 2 – nitrogen balance and whole tract digestibility

Six barren non-lactating cows (692±37.6 kg), were randomly allocated to either: standard PPO RC silage (RC+); low PPO RC silage (RC−) or PRG. All silages were offered at a fixed rate of 17 g DM/kg BW. The experiment consisted of two 3×3 Latin squares with each period 28 days, consisting of 16 days adaptation to the diet, 7 days recovery after gluing urine collection equipment and 5 days for N balance faecal and urine collection. First cut big bale silage for the RC was cut from the same plots as in experiment 1 on 26 May 2011 and second cut on 10 July 2011, grass was cut on 26 May 2011. Ensiling, animal weighing and feeding followed the same design as experiment 1.

### Sample collection methods – experiment 2

Feed sampling was as in experiment 1. Nitrogen balance was measured by collecting the total production of urine and faeces from each animal over a 5-day period, using externally applied urine and faeces separators (Moorby *et al*. 2009). Urine was preserved by acidification (1.4 litres of 2 M sulphuric acid added to each daily urine collection vessel). Subsamples of urine (1% of daily production) were stored at 4°C for each day and bulked across the 5-day total collection. Approximately 40 ml bulked urine per cow was sent fresh for analysis of total N concentration at the end of each period. Faeces were weighed and a representative sub-sample taken daily over the 5 days and oven-dried at 100°C for 48 h for determination of DM. A further sub-sample (3% of daily production) was stored at 4°C for each day and bulked across the 5 days total collection before being stored frozen before analysis.

### Chemical analysis

The same procedures as used by Lee *et al*. (2014) were used for chemical analysis of silages and digesta, in brief with the original reference in parentheses: water-soluble carbohydrate (WSC) was determined spectrophotometrically (Thomas, 1977); volatile fatty acids were determined by GLC; ammonia-N was assessed enzymatically using glutamate dehydrogenase on a discrete analyser; N was determined by a micro-Kjeldahl technique; fibre (NDF and ADF) were determined as described by Van Soest *et al*. (1991) and Van Soest and Wine (1967), respectively.

Chromium and ytterbium concentrations were analysed by atomic absorption spectrophotometry (Williams *et al*., 1962). Purine and pyrimidine bases were determined by HPLC (Cozzi *et al*., 1993). Amino acids (AAs) were determined by HPLC. Threonine and serine were corrected for loss (5% and 10%, respectively), whereas tryptophan and cysteine have been omitted due to degradation during acid hydrolysis. Fatty acids in silage were extracted and methylated as described by Sukhija and Palmquist (1988). Fatty acids in digesta were transesterified as described by Kramer and Zhou (2001) and analysed by GLC (Lee *et al*., 2014).

For the PPO activity, forage (ca. 0.5 g FW) was ground in liquid N before extracting in 2 ml of cooled McIlvaine buffer (McIlvaine, 1921) and following the procedure described by Lee *et al*. (2014). Total phenolics and fractions (hydroxycinnamates, isoflavonoids and flavonoids) were extracted from freeze-dried, ground forage tissue (125 mg) in methanol–acetic acid–water (85 : 0.5 : 14.5, v/v/v), and partitioned with ethyl acetate–ethyl ether (1 : 1, v/v), according to the method of Kagan and Flythe (2012). Protein-bound phenol formation in RC samples before baling was assessed using a modified Lowry procedure described by Winters and Minchin (2005).

## Statistical analysis of results

For experiment 1 background levels of chromium and ytterbium in digesta were considered before mathematical reconstitution of true digesta to calculate flow (correction factors (*R*) ranged between −0.1 and 0.1, reflecting representative estimation of flow; Faichney, 1975). Rumen pH and ammonia-N concentration were analysed using a repeated measure ANOVA (Genstat Release 13.2 (PC/Windows Vista)) with diet as the treatment effect, blocking per period plus animal and hourly sample as the repeated measure. Biohydrogenation of C18 PUFA was assessed as the difference between daily intake and duodenal flow (g/day) as a percentage of daily intake. Treatment *P*-values are reported along with SED of the treatment effect. For experiment 2, intake and faecal flow data were subjected to a general ANOVA with diet as the treatment effect and blocking per period plus animal. Tukey’s honest significant difference test was used post-ANOVA with significance stated at the *P*<0.05 level, whereas a trend was defined as *P*<0.1.

## Results

### Silage composition

The chemical compositions of experimental silages are given in Tables 1 and 2. For experiment 1 DM, organic matter (OM), WSC and fibre (ADF and NDF) were higher in PRG than either RC+ or RC−, which were comparable to each other. The RC silages had the highest total N over PRG, with RC− higher than RC+ (2 g/kg DM). Activity of PPO as cut was higher in RC+ than RC−, whereas total soluble phenolics were higher in RC− with little difference found in the RC as ensiled in PBP (9.92 g/kg DM). pH for all silages was high with PRG higher than the two RC silages. This was reflected in the fermentation acids which were lower in PRG than the two RC silages, which were comparable. Ammonia-N in the silages were higher in the two RC silages than PRG, with RC+ slightly higher than RC− (0.35 g/kg DM). Total and individual AA and fatty acid compositions of the silages are reported in Supplementary Tables S1 to S3.

**Table 1:** Chemical composition, polyphenol oxidase (PPO) activity and protein-bound phenol concentrations in the silages offered to cattle steers in experiment 1 (g/kg dry matter (DM) unless stated)

	RC+	RC−	PRG	SED
DM (g/kg fresh)	334	398	570	20.2
OM	904	900	941	1.7
Water-soluble carbohydrate	55.3	55.7	80.8	5.51
Total N	27.7	29.7	15.9	0.84
NDF	318	310	625	7.4
ADF	231	253	337	7.9
PPO activity (µkat^1^/g DM)	69.4	20.8	ND	3.68
Protein-bound phenol	10.2	9.64	ND	1.063
Hydroxycinnamates	0.04	0.12	ND	0.382
Isoflavonoids	3.39	1.59	ND	0.938
Flavonoids	0.40	0.12	ND	0.719
Total phenolics	3.83	1.83	ND	1.870
pH	4.27	4.19	5.49	0.052
Acetate	12.4	10.8	1.55	5.069
Propionate	0.53	0.31	0.09	0.206
*n*-Butyrate	0.90	0.28	0.03	0.306
*i*-Butyrate	0.76	0.37	0.10	0.399
*i*-Valerate	0.01	0.01	0.02	0.029
Ammonia-N	1.80	1.45	0.44	0.148

RC+=high polyphenol oxidase red clover silage; RC−=low polyphenol oxidase red clover silage; PRG=perennial ryegrass silage; OM=organic matter; ND=not detected.

1 Katal (SI unit of catalytic activity ~1 mol/s) of pre-ensiled material.

**Table 2:** Chemical composition, polyphenol oxidase (PPO) activity and protein-bound phenol concentrations in the silages offered dry dairy cattle in experiment 2 (g/kg dry matter (DM) unless stated)

	RC+	RC−	PRG	SED
DM (g/kg fresh)	231	264	377	8.3
OM	901	899	891	3.5
Water-soluble carbohydrate	15.3	13.3	195	20.5
Total N	31.1	34.1	26.9	0.70
NDF	410	389	395	2.2
ADF	319	298	223	13.9
PPO activity (µkat^1^/g DM)	48.3	10.5	ND	1.71
Protein-bound phenol	11.8	8.47	ND	1.163
Hydroxycinnamates	0.70	1.70	ND	0.059
Isoflavonoids	8.80	9.60	ND	1.145
Flavonoids	3.30	4.60	ND	0.053
Total phenolics	12.8	15.8	ND	2.357
pH	4.15	4.40	4.47	0.152
Acetate	15.4	15.1	6.86	4.980
Propionate	1.99	2.48	0.43	0.956
*n*-Butyrate	4.47	5.48	0.23	2.533
*i*-Butyrate	1.28	1.98	0.17	0.856
*i*-Valerate	0.18	0.11	0.12	0.164
Ammonia-N	1.96	2.94	2.07	0.473

RC+=high polyphenol oxidase red clover silage; RC−=low polyphenol oxidase red clover silage; PRG=perennial ryegrass silage; OM=organic matter; ND=not detected.

1 Katal (SI unit of catalytic activity ~1 mol/s).

For experiment 2 the silages were much wetter than in experiment 1 and the differences between PRG and RC silages less prominent for DM, OM and fibre, whereas the difference in WSC was greater. Total N was higher for the RC silages than PRG but the differential was smaller than in experiment 1 (32.5 *v*. 26.9 g/kg DM, between RC silage and PRG, respectively). Polyphenol oxidase activity in the as cut RC forage was higher on RC+ than RC− for both experiments. Total phenolics in experiment 1 were higher in RC+ than RC− in contrast to experiment 2 where total phenolics were higher in RC−. Protein-bound phenols were higher in RC+ than RC− in the as-ensiled samples. pH for all silages were high with little difference between silages (4.34). Fermentation acids were lower in PRG than the RC silages which were comparable to each other. Ammonia-N for all silages were similar with RC− slightly higher than the other two silages.

### Rumen parameters – experiment 1

**Figure 1:**
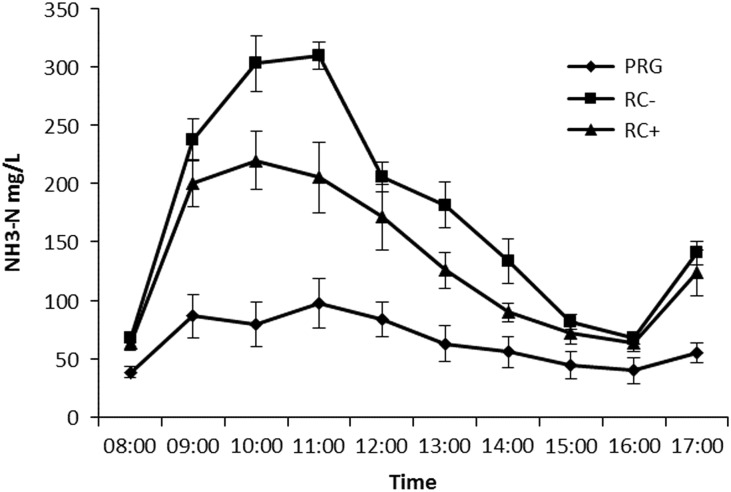
Temporal rumen ammonia-N (NH_3_-N) concentrations in steers offered the three experimental silages in experiment 1. PPO=polyphenol oxidase; RC+=high PPO red clover silage; RC−=low PPO red clover silage; PRG=perennial ryegrass silage.

Mean rumen pH was highest for RC silages than PRG (Table 3). Temporal rumen ammonia-N is shown in Figure 1 with ammonia-N increasing following the 0800 h feeding to reach a peak at 1000 for RC− of 22 mg N/l and 1100 for RC+ of 309 mg N/l and PRG of 97.5 mg N/l. Levels then declined to pre-feeding levels by 1600 before increasing following feeding. The PRG was lower than both RC silages and significant at all time points. High PPO red clover silage was lower than RC− at 1000, 1100, 1300 and 1400. Mean rumen ammonia-N across the day is reported in Table 3 with a higher concentration in RC− silage fed animals compared to either RC+ or PRG, which in turn was lowest for PRG. Biohydrogenation of C18 PUFA is reported in Table 3 with both C18:2n-6 and C18:3n-3 showing the same pattern with both RC silages lower than PRG, but with no difference between RC− and RC+.

**Table 3:** Rumen parameters in steers fed the three silage diets in experiment 1

Item	RC+	RC−	PRG	SED	*P*-value
pH^1^	6.80^b^	6.74^b^	6.51^a^	0.051	**
Ammonia-N (mg N/l)^2^	133^b^	173^c^	64.5^a^	11.03	***
C18:2 Biohydrogenation^3^	75.8^a^	75.1^a^	82.8^b^	1.65	**
C18:3 Biohydrogenation^3^	77.0^a^	77.4^a^	91.1^b^	1.31	***

RC+=high polyphenol oxidase red clover silage; RC−=low polyphenol oxidase red clover silage; PRG=perennial ryegrass silage.

^a,b,c^ Means within row not bearing a common letter differ (*P*<0.05).

1 Time (*P*<0.001); treatment×time (NS). Treatment SED and *P*-value reported.

2 Time and treatment×time (*P*<0.001). Treatment SED and *P*-value reported.

3 Biohydrogenation percentage difference between intake and duodenal flow.

**P<0.01 and ***P<0.001.

### Intake, duodenal flow and digestibility – experiment 1

Dry matter and OM intake were comparable across diets (9.14 and 8.36 kg/day, respectively), whereas total N intake was highest on RC− followed by RC+ and lowest on PRG (Table 4). Other than alanine the same pattern as for total N was seen for intake of total and individual AAs. For alanine RC+ and RC− were similar and both higher than PRG.

**Table 4:** Intake and duodenal flow of N and amino acids (AA) in steers fed the three silage diets in experiment 1 (g/day, unless stated)

Item	RC+	RC−	PRG	SED	*P*-value^8^
Intake					
DM (kg/day)	9.26	9.40	8.76	0.344	NS
OM (kg/day)	8.37	8.46	8.24	0.323	NS
Total N	256^b^	279^c^	138^a^	6.7	***
AAs					
Aspartic acid	209^b^	230^c^	52.5^a^	6.40	***
Threonine	48.9^b^	52.7^c^	24.5^a^	1.22	***
Serine	49.7^b^	54.0^c^	22.8^a^	1.16	***
Glutamic acid	96.1^b^	107^c^	39.5^a^	2.32	***
Proline	92.8^b^	110^c^	40.9^a^	1.82	***
Glycine	46.8^b^	50.8^c^	26.3^a^	1.31	***
Alanine	63.8^b^	67.7^b^	38.1^a^	1.71	***
Valine	65.0^b^	70.6^c^	34.0^a^	1.60	***
Methionine	13.0^b^	14.2^c^	8.57^a^	0.404	***
Isoleucine	53.5^b^	57.7^c^	26.7^a^	1.37	***
Leucine	86.8^b^	94.9^c^	45.6^a^	2.37	***
Tyrosine	28.0^b^	31.9^c^	11.1^a^	1.29	***
Phenylalanine	53.9^b^	58.8^c^	27.6^a^	1.44	***
Histidine	22.9^b^	24.6^c^	9.84^a^	0.602	***
Lysine	73.3^b^	78.6^c^	35.2^a^	1.77	***
Arginine	36.4^b^	43.3^c^	20.9^a^	1.54	***
Total AA (kg/day)	1.04^b^	1.15^c^	0.46^a^	0.027	***
Duodenal flow					
DM (kg/day)	6.42	6.55	6.21	0.437	NS
OM (kg/day)	5.05	5.21	5.13	0.389	NS
OMAD (%)	39.6	38.3	37.8	3.48	NS
OMTD (%)	55.4	55.8	51.5	2.43	NS
Total N	280^b^	291^b^	197^a^	15.3	***
Microbial N	116	130	101	13.0	NS
Non-microbial N^1^	164^b^	161^b^	96.4^a^	6.57	***
AAs					
Aspartic acid	112^b^	125^b^	82.5^a^	8.17	**
Threonine	51.4^b^	57.3^b^	38.7^a^	3.70	**
Serine	46.4^b^	51.4^b^	32.6^a^	3.26	***
Glutamic acid	122^b^	135^b^	90.8^a^	8.96	**
Proline	44.4^b^	48.9^b^	31.6^a^	3.02	***
Glycine	71.3^b^	78.4^b^	46.7^a^	5.57	***
Alanine	61.0^b^	67.5^b^	47.0^a^	4.57	**
Valine	60.5^b^	66.9^b^	43.5^a^	4.29	**
Methionine	20.2^b^	22.4^b^	15.9^a^	1.66	*
Isoleucine	53.8^b^	60.0^b^	39.5^a^	3.93	**
Leucine	84.1^b^	94.2^b^	59.3^a^	5.82	***
Tyrosine	39.7^b^	44.5^b^	25.5^a^	3.47	**
Phenylalanine	56.0^b^	63.0^b^	39.7^a^	3.93	***
Histidine	23.0^b^	25.8^b^	15.0^a^	1.57	***
Lysine	72.0^b^	80.7^b^	54.6^a^	5.46	**
Arginine	47.2^b^	53.8^b^	32.1^a^	3.79	***
Total AA (kg/day)	0.97^a^	1.08^b^	0.70^a^	0.070	**
Microbial AA^2^	0.51	0.57	0.44	0.057	NS
Non-microbial AA^3^	0.46^b^	0.51^b^	0.25^a^	0.039	***
EMPS^4^	35.6	41.4	33.1	6.19	NS
EMPS^5^	25.2	27.8	23.8	3.08	NS
MN/NI^6^ (%)	45.2^a^	46.7^a^	72.3^b^	6.49	**
NMN/NI^7^ (%)	63.8	57.9	70.3	5.29	NS

RC+=high polyphenol oxidase red clover silage; RC−=low polyphenol oxidase red clover silage; PRG=perennial ryegrass silage; DM=dry matter; OM=organic matter; OMAD=organic matter apparently digested; OMTD=organic matter truly digested; NS=not significant; EMPS=efficiency of microbial protein synthesis; MN=microbial N; NI=nitrogen intake.

^a,b,c^ Means within row not bearing a common letter differ (*P*<0.05).

1 Combination of dietary and ammonia-N.

2 Total microbial AA (microbial N×6.25×0.7; Hvelplund, 1986) – kg/day.

3 Total non-microbial AA (total AA – total microbial AA) – kg/day.

4 Efficiency of microbial protein synthesis, g microbial N/kg OMAD in the rumen.

5 Efficiency of microbial protein synthesis, g microbial N/kg OMTD in the rumen.

6 Microbial N at the duodenum as a percentage of N intake.

7 Non-microbial N (dietary+ammonia+endogenous) at the duodenum as a percentage of N intake.

8 NS=NS at *P*<0.05.

* *P*<0.05, **P<0.01 and ***P<0.001.

Duodenal flow of DM, OM and microbial N were comparable for all three silage diets (6.39, 5.13 and 0.116 kg/day, respectively) as were organic matter apparently digested (OMAD) and organic matter truly digested (OMTD) (38.6% and 54.2%, respectively). Total N, non-microbial nitrogen (NMN), total and individual AA duodenal flow was higher for the two RC silages than PRG with no difference between RC+ and RC−. Flow of microbial AA as calculated by Hvelplund (1986) were not different between treatments averaging 0.51 kg/day. Non-microbial AA flow was higher for the two RC silages compared to PRG with no difference between RC+ and RC−. Efficiency of microbial protein synthesis calculated either using OMAD or OMTD were not different between treatments averaging 36.7 and 25.6 g microbial N/kg OMAD or OMTD, respectively. Microbial N flow as a percentage of total N intake was significantly higher on PRG as opposed to the two RC silages, with no difference between RC+ and RC−. The NMN flow as a percentage of total N intake was not different across the three silage diets averaging 64% (Table 4).

Total fatty acid, sum of C18:1 *cis*, sum of conjugated linoleic acid, C18:2n-6 and C18:3n-3 duodenal flow was highest in RC−, followed by RC+ and lowest in PRG. For the sum of C18:2 non-conjugated dienes (excluding C18:2n-6) both RC silages resulted in similar flows which were higher than PRG. Similarly, C18:0 was comparable between RC silages and lower in PRG when compared with RC−. The sum of C18:1 *trans* was highest in RC− and lowest in RC+ and PRG which were comparable. C12:0 and C14:0 duodenal flow was highest on the PRG diet with both RC silage diets comparable. No significant difference was observed in the duodenal flow of C16:0, C16:1n-7, phytanic acid, C20:0 or branched and odd chain (BOC) fatty acids (Table 5).

**Table 5:** Intake and duodenal flow (g/day, unless otherwise stated) of dry matter (DM) and fatty acids in steers fed the three silage diets

	RC+	RC−	PRG	SED	*P*-value^4^
Intake					
DM (kg/day)	9.26	9.40	8.76	0.344	NS
Fatty acids					
C12:0	0.28^a^	0.30^a^	0.47^b^	0.017	***
C14:0	0.70^a^	0.79^a^	1.05^b^	0.043	***
C16:0	31.0^b^	34.2^c^	25.7^a^	1.02	***
C16:1n-7	0.26^a^	0.20^a^	0.47^b^	0.05	***
C18:0	4.56^b^	4.64^b^	2.84^a^	0.144	***
C18:1n-9	4.02^a^	4.78^b^	4.21^a^	0.179	**
C18:2n-6	35.6^b^	39.3^c^	22.7^a^	1.06	***
C18:3n-3	70.8^b^	84.5^c^	59.7^a^	2.27	***
C20:0	1.71^b^	1.84^b^	1.22^a^	0.070	***
ΣBOC^1^	2.30^b^	2.32^b^	1.63^a^	0.156	**
Total	165^b^	189^c^	129^a^	5.2	***
Duodenal flow					
DM (kg/day)	6.42	6.55	6.21	0.437	NS
Fatty acids					
C12:0	0.50^a^	0.50^a^	0.77^b^	0.069	**
C14:0	2.37^a^	2.38^a^	3.32^b^	0.257	**
C16:0	43.3	48.4	42.3	2.38	NS
C16:1n-7	0.28	0.29	0.26	0.029	NS
Phytanic acid	5.38	5.88	5.75	0.385	NS
C18:0	116^ab^	131^b^	111^a^	6.7	*
ΣC18:1*cis*	8.86^b^	10.5^c^	6.33^a^	0.572	***
ΣC18:1trans	12.2^a^	17.0^b^	11.8^a^	0.83	***
ΣCLA^2^	1.50^b^	1.67^c^	0.43^a^	0.058	***
ΣC18:2 NC^3^	3.01^b^	3.82^b^	2.14^a^	0.780	**
C18:2n-6	8.58^b^	9.76^c^	3.93^a^	0.487	***
C18:3n-3	16.2^b^	19.0^c^	5.34^a^	0.782	***
C20:0	3.36	3.51	3.09	0.204	NS
ΣBOC^1^	25.1	25.1	27.0	1.17	NS
Total	261	294	233	14.1	**

RC+=high polyphenol oxidase red clover silage; RC−=low polyphenol oxidase red clover silage; PRG=perennial ryegrass silage; NS=not significant; NC=non-conjugated; BOC=branched and odd chain.

^a,b,c^ Means within row not bearing a common letter differ (*P*<0.05).

1 BOC fatty acids (sum of C11:0, C15:0, C15 iso, C15 ante, C15:1, C17:0, C17 iso, C17 ante, C17:1 and C19:0).

2 Sum of all isomers of conjugated linoleic acid.

3 Sum of all NC C18:2 (other than C18:2n-6).

4 NS=NS at *P*<0.05.

*P<0.05, **P<0.01 and ***P<0.001.

Proportions of C18:1 isomers (*cis* and *trans*) in the duodenal digesta of steers offered the three silage diets are reported in Table 6. For *cis* isomers, *cis*-9 was the most dominant averaging 77.7% of all *cis* isomers, with PRG showing the highest proportion followed by RC− and lowest in RC+. *cis*-11 proportion of total *cis* was similar between PRG and RC+ and lowest in RC−, whereas the opposite relationship was exhibited for *cis*-14. *cis*-12 and *cis*-13 showed a higher proportion on both RC silage diets than PRG and *cis*-15 was comparable across all diets contributing <1% of total *cis* isomers. *trans*-11 was the most dominant *trans* isomer averaging 42.4%, with PRG significantly higher than both RC silages which were comparable. For *trans*-10, -12, -13_14 and -16 the opposite relationship was found with both RC silages showing higher proportions than PRG. *trans*-15 was highest on RC− followed by RC+ and lowest on PRG. No difference in the proportions of *trans*-4, -5, -6_7_8 and -9 were found.

**Table 6:** Proportions (%) of C18:1 isomers in the duodenal digesta of steers fed the three silage diets

	RC+	RC−	PRG	SED	*P*-value^3^
C18:1 *cis*					
9	75.7^a^	77.9^b^	79.6^c^	0.71	**
11	13.2^b^	10.8^a^	12.6^b^	0.43	***
12	5.84^b^	5.72^b^	3.12^a^	0.308	***
13	1.96^b^	1.99^b^	1.49^a^	0.166	*
14	2.77^b^	3.15^c^	2.35^a^	0.167	**
15	0.59	0.46	0.91	0.210	NS
C18:1 trans					
4	0.24	0.30	0.11	0.117	NS
5	0.47	0.40	0.39	0.069	NS
6_7_8^1^	3.48	3.45	3.53	0.062	NS
9	2.43	2.42	2.60	0.085	NS
10	4.37^b^	4.41^b^	3.85^a^	0.108	***
11	38.8^a^	34.4^a^	53.9^b^	1.35	***
12	7.10^b^	7.99^b^	5.48^a^	0.151	***
13_14^1^	12.8^b^	13.8^b^	8.16^a^	0.438	***
15^2^	12.9^b^	14.3^c^	9.26^a^	0.401	***
16	17.4^b^	18.6^b^	12.7^a^	0.66	***

RC+=high polyphenol oxidase red clover silage; RC−=low polyphenol oxidase red clover silage; PRG=perennial ryegrass silage; NS=not significant.

^a,b,c^ Means within row not bearing a common letter differ (*P*<0.05).

1 Unresolved peaks reported together.

2 Reported as combined peak with C18:1 *cis*-10, which could not be resolved.

3 NS=NS at *P*<0.05

*P<0.05, **P<0.01 and ***P<0.001.

### Intake, faecal flow and digestibility – experiment 2

Dry matter and OM intake were comparable across the three silage diets averaging 12.8 and 11.6 kg/day, respectively (Table 7). Total N, total AA, aspartic acid and valine intake were highest on RC− followed by RC+ and lowest on PRG. Intake of threonine, serine, isoleucine, leucine, phenylalanine, histidine and arginine were higher on the RC silages than the PRG but with no difference between RC+ and RC−. For glutamic acid, glycine and alanine intake on RC− was higher than PRG but there was no difference between RC+ and PRG or RC+ and RC−. Intake of methionine was higher for PRG than the RC silages with no difference between RC+ and RC−. Intake of tyrosine was higher for RC− than the other silages, whereas lysine was lowest with RC+ than PRG, with no difference to RC−. Intake of proline was comparable across the three silage diets.

**Table 7:** Intake and faecal output of N and amino acids (AA) in steers fed the three silage diets in experiment 2 (g/day, unless stated)

Item	RC+	RC−	PRG	SED	*P*-value^1^
Intake					
DM (kg/day)	12.8	12.4	13.1	0.30	NS
OM (kg/day)	11.6	11.3	11.9	0.27	NS
Total N	369^b^	426^c^	353^a^	11.0	***
AAs					
Aspartic acid	173^b^	203^c^	128^a^	7.4	***
Threonine	69.1^b^	73.4^b^	62.6^a^	2.01	**
Serine	60.2^b^	60.6^b^	55.4^a^	1.77	*
Glutamic acid	132^ab^	141^b^	125^a^	4.5	*
Proline	84.3	88.1	91.5	4.68	NS
Glycine	70.2^ab^	74.2^b^	66.7^a^	2.24	*
Alanine	98.8^ab^	101^b^	93.2^a^	3.15	tr
Valine	91.8^b^	98.3^c^	82.5^a^	2.56	***
Methionine	21.4^a^	21.7^a^	24.3^b^	0.84	*
Isoleucine	77.6^b^	82.7^b^	67.9^a^	2.18	***
Leucine	130^b^	139^b^	118^a^	3.66	***
Tyrosine	32.4^a^	37.9^b^	30.5^a^	1.49	**
Phenylalanine	84.7^b^	89.0^b^	79.0^a^	2.43	*
Histidine	35.2^b^	36.9^b^	28.8^a^	1.09	***
Lysine	84.6^a^	90.2^ab^	92.0^b^	2.54	*
Arginine	43.2^b^	47.2^b^	30.8^a^	1.63	***
Total AA (kg/day)	1.29^b^	1.38^c^	1.18^a^	0.041	**
Faecal output					
DM (kg/day)	4.7^b^	4.8^b^	3.6^a^	0.11	***
OM (kg/day)	4.0^b^	4.1^b^	2.8^a^	0.09	***
OMAD (%)	65.3^a^	63.6^a^	76.4^b^	0.68	***
Total N	136^b^	150^c^	118^a^	4.3	***
AAs					
Aspartic acid	50.9^ab^	53.9^b^	46.8^a^	1.82	**
Threonine	25.1^ab^	26.3^b^	23.4^a^	0.93	*
Serine	23.8^b^	25.3^b^	18.9^a^	0.89	***
Glutamic acid	54.8	56.9	53.6	2.02	NS
Proline	27.3^b^	28.9^b^	21.9^a^	0.88	***
Glycine	29.0^b^	31.2^b^	24.8^a^	1.02	***
Alanine	32.1	33.7	33.6	1.61	NS
Valine	29.9^ab^	32.0^b^	26.8^a^	1.03	**
Methionine	9.33^a^	9.95^ab^	10.6^b^	0.400	*
Isoleucine	26.5^ab^	28.5^b^	24.8^a^	0.86	**
Leucine	40.9^b^	43.6^b^	35.3^a^	1.45	***
Tyrosine	17.2^ab^	19.1^b^	15.9^a^	0.90	*
Phenylalanine	29.4^b^	31.5^b^	24.5^a^	0.982	***
Histidine	13.1^b^	14.3^b^	9.89^a^	0.494	***
Lysine	32.7	35.0	32.7	1.22	NS
Arginine	20.9^ab^	21.9^b^	18.9^a^	0.90	*
Total AA (kg/day)	0.46^ab^	0.49^b^	0.42^a^	0.017	**

RC+=high polyphenol oxidase red clover silage; RC−=low polyphenol oxidase red clover silage; PRG=perennial ryegrass silage; OM=organic matter; NS=not significant; OMAD=organic matter apparently digested.

^a,b,c^ Means within row not bearing a common letter differ (*P*<0.05).

1 NS=NS at *P*<0.05; tr=trend *P*<0.1.

*P<0.05, **P<0.01 and ***P<0.001.

Faecal output of DM and OM was higher for the two RC silages than PRG with no difference between RC+ and RC−. Likewise, OMAD was higher for PRG than the RC silages. Total N faecal output was highest for RC− followed by RC+ and lowest in PRG. Faecal AA serine, proline, glycine, leucine, phenylalanine and histidine were higher from RC silage diets than PRG with no difference between RC+ and RC−. Faecal output of aspartic acid, threonine, valine, isoleucine, tyrosine, arginine and total AA were higher on RC− than PRG but there was no difference between RC− and RC+ and RC+ and PRG. Faecal output of glutamic acid, alanine and lysine were comparable across the three silage diets (Table 7).

**Figure 2:**
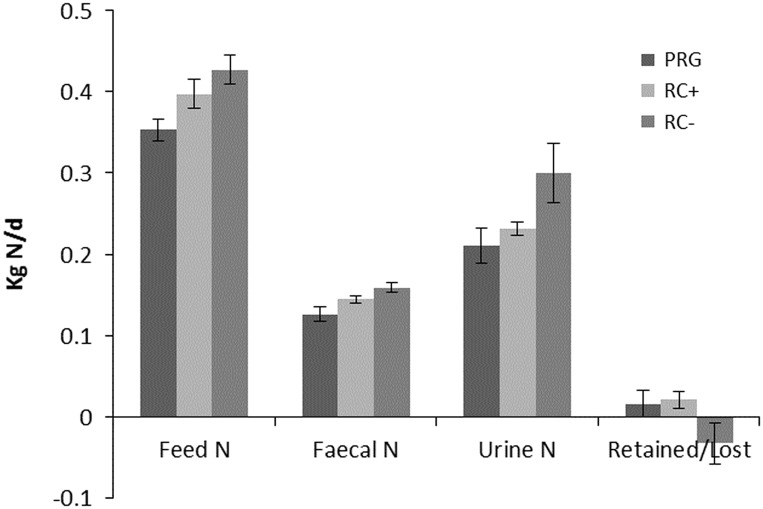
Nitrogen balance (kg/day) in cattle offered the three experimental silages in experiment 2. PPO=polyphenol oxidase; RC+=high PPO red clover silage; RC−=low PPO red clover silage; PRG=perennial ryegrass silage.

**Figure 3:**
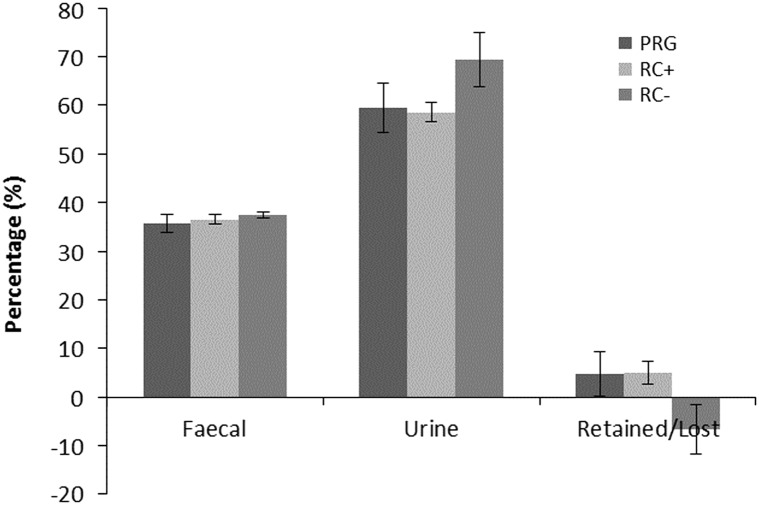
Percentage of dietary nitrogen in faeces, urine and retained in cattle offered the three experimental silages in experiment 2. PPO=polyphenol oxidase; RC+=high PPO red clover silage; RC−=low PPO red clover silage; PRG=perennial ryegrass silage.

Nitrogen balance as kg/day and percentage of N intake are shown in Figures 2 and 3, respectively. Urinary N loss as kg/day and percentage of total N intake was significantly higher in RC− than either RC+ and PRG, which were not different from each other. Faecal N loss as a proportion of N intake was comparable across the three silage diets. Total N balance ((urine N+faecal N)/intake N) as N retained or lost was comparable for PRG and RC+ which showed a small N gain and were higher than RC− which showed a loss of N over N intake. Differences in cow BW across periods were small and did not reflect any difference between treatments.

## Discussion

### Silage composition

Silage composition in relation to quality is included in the Supplementary Material S2. Polyphenol oxidase activity, phenolics and PBP were not measured in PRG as it has been previously shown that PRG has little PPO and grass PPO does not offer protection to protein and C18 PUFA in the rumen (Lee *et al*., 2014). Polyphenol oxidase and free phenolics were assessed in the RC as cut before wilting as it is known that after plant cell damage (cutting and wilting) rapid deactivation of PPO occurs by quinone binding formed through the PPO-catalysed conversion of phenolic substrate. Lee *et al*. (2013) reported a rapid decline in PPO activity in macerated RC within 1 h with the rate relating to the extent of damage of the crop. Winters *et al*. (2008) has previously reported a differential of between×8 and 38 in PPO activity between the two cultivars (RC+ and RC−) used in the current study across a season. However, in the current study a differential of only×3.3 and 4.6 were found for experiments 1 and 2, respectively. This may relate to a difference in sampling technique, as only foliar samples were taken in the study of Winters *et al*. (2008) and immediately flash frozen in liquid N. Equally it could also relate to a change in gene activity during seed multiplication as it is known that RC has up to six PPO genes which express different levels of activity anatomically and seasonally within the plant (Winters *et al*., 2009).

The phenolic compounds identified within RC samples are those identified previously as the major groups in RC namely: isoflavonoids, being the most abundant, followed by flavonoids and hydroxycinnamates (Parveen *et al*., 2010; Kagan *et al*., 2016). Although hydroxycinnamates are the lowest in concentration the group contains phasleic acid and clovamide, which are the *o-*diphenolic substrate for RC PPO. Phasleic acid content in the RC cultivars used here was shown to vary across the season and between the low and high PPO cultivars, with no clear pattern. Although reported on a FW basis, if we assume a DM of 20% to compare with the current study, Winters *et al*. (2008) reported a range of 5 to 18.8 g/kg DM compared to the present range across the two experiments of 0.04 to 1.70 g/kg DM for total hydroxycinnamates. As with PPO, this could relate to the difference in sampling as previous studies have reported a rapid loss during wilting and a greater proportion in foliar tissue (Lee *et al*., 2009; Kagan *et al*., 2016). Isoflavonoids have been linked with decreased fertility in sheep (Braden *et al*., 1967), and reduced action of hyper ammonia-producing bacteria (HAB) in cattle and goats (Flythe and Kagan, 2010; Flythe *et al.*, 2013) and although not a substrate for PPO have been associated with non-enzymatic protein binding, which could increase PBP (Kagan *et al*., 2016). In experiment 1, there was no difference in PBP content taken after wilting between RC+ and RC−, whereas in experiment 2 RC+ was greater than RC−, as one may expect due to differences in PPO activity. Although PBP is a useful indicator of PPO induced protection through protein binding, Lee *et al*. (2014) noted that as complexing continues protein solubility will be reduced which will reduce the accuracy of the assay which only detects soluble protein. The similar PBP in experiment 1 could be related to the higher hydroxycinnamates in RC− which have also been shown to induce non-enzymatic protein complexing (Lee *et al*., 2013). As PPO complexing of protein has been reported to reduce proteolysis in silo and so reduce loss of protein as ammonia. The higher levels of ammonia-N as a proportion of total N (0.09) in RC− in experiment 2 may be related to a reduced protein complexing through PPO or a combination of an extended wilt and the higher pH of the silage resulting in greater protease activity increasing proteolysis.

### Dry matter intake and ruminal pH

In the current study, all silages were limited to 14 to 15 and 17 g DM/kg BW daily, in experiments 1 and 2, respectively, which should equate to a range of intakes between 8.20 to 9.85 and 11.1 to 12.4 kg DM/day, for experiments 1 and 2, respectively. Dry matter intakes in experiment 1 fell within the range and were not different to each other, and whilst RC+ and PRG were slightly greater than the range, possibly due to underestimation of DM content, no difference was also observed across dietary intake.

Rumen pH mean values relate to values reported on similar high DM and fibre content RC and PRG (Lee *et al*., 2014), but were higher than those reported previously on sole forage diets (Merry *et al*., 2006). In the present study unlike Lee *et al*. (2014), there was no significant interaction effect (treatment×diet) for ruminal pH with the silage diets. This may reflect similar eating patterns across diets, whereas Lee *et al*. (2014) reported a grazing consumption pattern for grass across the day with most RC consumed in one session.

### Nitrogen and amino acid metabolism across the rumen

Ammonia-N in the rumen was significantly higher on RC silage compared to PRG diets reflecting N intake; when corrected for intake (rumen ammonia-N/N intake) the same pattern is observed with RC− 0.67, RC+ 0.51 and PRG 0.47. Numerous studies with RC have shown a significant reduction of rumen ammonia-N release per unit of dietary N consumed compared to other forages reflecting a potential protective nature of PPO driven protein binding reducing solubility and degradability in the rumen (Dewhurst *et al*., 2003; Merry *et al*., 2006; Vanhatalo *et al*., 2009). In the current study, RC+ had a lower ratio of rumen ammonia-N per unit of dietary N than RC− but both were higher than PRG. Lee *et al*. (2014) when feeding RC and PRG reported values of 0.62 and 0.78, respectively. The current PRG ratio may reflect the low N content of the grass (ca. 10% CP). The lower ratio of RC+ may reflect a level of PPO protection or an isoflavanoid impact of HAB Flythe and Kagan (2010) as both were higher on RC+ compared to RC−. However, if this difference between RC+ and RC− was PPO driven it was despite comparable PBP values, which although as already described does not reflect total binding (due to loss of solubility), it may also indicate the difference is more related to dietary N intake being higher than microbial dietary N capture potential on RC− than any PPO related protection. This lack of difference in protein complexing, predicted by PBP, may also explain the comparable PUFA biohydrogenation results observed (discussed later).

Efficiency of microbial protein synthesis values across the treatments were not significantly different when reported as a proportion of OMAD or OMTD, averaging 36.7 and 25.6, respectively, which is comparable to previous values of 35.8 and 21.7 as an average of animals offered RC and PRG, which were also non-significantly different (Lee *et al*., 2014). Values of OMAD and OMTD were comparable across treatments and similar to the study of Lee *et al*. (2014), although lower than the meta-analysis of typical North American and European ruminant diets performed by Huhtanen *et al*. (2010) which predicted values of 42% and 74%, respectively, for OMAD and OMTD.

Higher total N duodenal flows with RC silage than PRG agrees with previous results comparing RC with grass (Dewhurst *et al*., 2003; Merry *et al*., 2006; Vanhatalo *et al*., 2009; Lee *et al*., 2014), and is related to the greater N content of the RC. Total N flow with both RC diets was composed mainly of NMN (dietary, endogenous and ammonia-N) with the contribution of microbial N lower than with PRG as previously reported (Dewhurst *et al*., 2003; Merry *et al*., 2006; Vanhatalo *et al*., 2009: Lee *et al*., 2014). These authors propose that the higher NMN with RC silage is due to a lower protein degradability in the rumen attributed to PPO activity protecting dietary protein (Lee *et al*., 2014; Merry *et al*., 2006; Halmemies-Beauchet-Filleau *et al*., 2014). However, no difference was observed between RC+ and RC− even when reported as a percentage of N intake. This would suggest a similar level of protein degradability in the rumen between the high and low PPO RCs and therefore a comparable level of protein complexing as inferred by the similar PBP content in the pre-ensiled samples. The comparable PBP between RC+ and RC−, despite different levels of PPO activity, may have been due to non-enzymatic complex driven binding of the substrate as previously reported (Lee *et al*., 2013; Kagan *et al*., 2016). The lack of difference between RC silages and PRG may be related to the greater proportional increase in N flow compared to N intake on PRG (1.42) compared to RC silages (1.33 and 1.27, for RC+ and RC−, respectively), possibly due to greater N recycling on the grass treatments; however, they may also indicate a degree of over estimation in duodenal flow as also reported by Lee *et al*. (2014). The calculated flow of AA within the NMN fraction indicates a greater flow of dietary protein on the RC treatments compared to PRG, with no difference between RC+ and RC−. The current study therefore showed little evidence for any difference between RC+ and RC− for dietary N protection, despite their different PPO activities, as assessed by either the proportion of NMN/N intake or the total NMN AA duodenal flow.

Patterns of AA flow at the duodenum are like those previously reported when consuming grass and RC silage (Vanhatalo *et al*., 2009; Lee *et al*., 2014) and further emphasise the limiting amount of histidine and methionine on all forage diets.

### Nitrogen balance

The chemical composition of the silages used for the N balance study were different to those used for the assessment of N metabolism across the rumen. Nitrogen intakes were higher in experiment 2 than experiment 1, although the pattern was the same of RC−>RC+>PRG. Organic matter apparently digested for PRG and RC silages were similar to those reported by Moorby *et al*. (2009) for grass silage (72.8) and RC silage (64.1). Apparent total AA digestibility was also comparable to previously reported values of 55% and 67%, respectively, for grass and RC silage in dairy cows reported by Lee *et al*. (2009b), with an average across forages in the current study of 64%, with no difference between treatments. In the study of Lee *et al*. (2009b) except for proline, where there was no difference and methionine which showed the opposite response, all other AA had a greater whole tract digestibility on RC than PRG. In the current study (data not shown), most AA whole tract digestibilities were comparable across treatments except for arginine and aspartic acid which were higher on the RC silages, and glycine, histidine, phenylalanine, proline and serine which were higher on PRG, which may reflect the higher total N content of the grass silage in the current study than Lee *et al*. (2009b); 26.9 *v*. 22.1 g/kg DM, respectively. It was previously reported that PPO induced complexing would reduce digestibility of AA which are likely to be phenol binding sites, for example, sulphur-containing AA (Lee *et al*., 2003, 2009 and 2014). Despite showing higher levels of PBP in RC+ (issues regarding PBP, have already been discussed), the lack of a digestibility differences of sulphur-AA between RC+ and RC− reflects similar protein complexing between the two silages, and may explain the small difference in experiment 2 between RC+ and RC−. Daily output of N in faeces and urine mirrored intake as also reported by Moorby *et al*. (2009) and Lee *et al*. (2009b). As a proportion of N intake, faecal N was comparable across treatments as previously reported with a mean of 32% and 38% for Moorby *et al*. (2009) and Lee *et al*. (2009b), respectively. Proportion of N intake as urinary N was also comparable in the previous studies averaging 40% and 43% (both studies were on lactating animals, hence the lower proportion compared to the current study), whereas in the current study RC− was significantly higher than PRG and RC+ which may reflect a level of PPO protection or indicative of the difference in N intake. Similarly, N retained in the current study showed no difference between RC+ and PRG but was significantly higher than RC−. The lack of difference between RC+ and PRG may have been due to the high level of WSC in the grass silage resulting in an elevation in the NUE of the grass diet as a consequence of the greater balance of N and energy in the rumen (Miller *et al*., 2001). However, previous studies with RC silage have also failed to show an elevation in NUE compared with grass or white clover silages (Bertilsson and Murphy, 2003; Van Dorland *et al*., 2006 and 2007). The variation in the response to RC across different studies is associated with N intake (Lee, 2014). Poppi and McLennan (1995) reported that N losses occur in grasses and legumes when CP content exceeds 210 g/kg of digestible OM. In the present study, these values were 303, 370 and 242 g CP/kg of digestible OM (intake OM – faecal OM) for RC+, RC− and PRG, respectively. Therefore, even if there was any advantage in NUE inferred by the RC’s PPO it would be lost due to the excessive N input (Lee, 2014). Reducing N pollution in urine is a critically important consideration for sustainable livestock production. Lowering CP intake from 200 to 150 g/kg DM could reduce N in urine by 66%, these values compare with 180, 214 and 168 g/kg DM for RC+, RC− and PRG in the current study and highlights the need to consider total N intake to optimise NUE which will otherwise counteract any potential benefits of PPO.

### Fatty acid metabolism across the rumen

Net gain of fatty acids between intake and duodenum have previously been reported on low lipid all forage diets and are consistent with microbial fatty acid synthesis *de novo* (Lee *et al*., 2006a; Halmeimies-Beauchet-Filleau *et al*., 2013; Lee *et al*., 2014). The response was greater with PRG as exemplified by a higher proportional increase in fatty acids associated with microbial lipids namely C16:0 and BOC (Vlaeminck *et al*., 2006), as also reported by Lee *et al*. (2014) on grass silage.

Biohydrogenation is the process where C18 unsaturated fatty acids are saturated by rumen bacteria; however, the process is often not complete, producing a range of intermediate C18:1 and C18:2 *trans* and *cis* isomers as exemplified in the duodenal digesta. Red clover has been shown to significantly reduce biohydrogenation as reviewed by Buccioni *et al*. (2012), with PPO implicated as a mechanism of action. However, in contrast to our hypothesis the high PPO RC+ did not result in a greater protection of PUFA across the rumen over RC−. The mechanism by which RC protects PUFA across the rumen is not well understood with a review analysing potential mechanisms published by Lee (2014). These mechanisms included entrapment of lipid within protein complexes as a consequence of PPO activity, alterations in digestion kinetics and ruminal microbial ecology. The current study suggests that PPO has little effect on biohydrogenation as RC+ failed to show any protection of PUFA compared to RC−. There is little doubt that PPO in both RC and grasses can deactivate lipase activity (Lee *et al*., 2006b; Van Ranst *et al*., 2011). However, for PPO to protect lipid in the rumen environment there appears to be a requirement for the lipid to be complexed within PBP which would limit microbial lipase access to the PUFA–glycerol ester bond (Lee *et al*., 2010). This requirement for the formation of a protein lipid emulsion protected by PBP complexing was confirmed by Gadeyne *et al*. (2015). They protected C18 PUFA rich oils through forming an emulsion with a protein extract of RC through the addition of a diphenolic substrate (4-methylcatechol). In the current study, the level of PBP (an indicator of complexing) was comparable between RC+ and RC− which may suggest a similar level of lipid PBP complexing. The ability to complex protein is driven by enzyme activity, protein AA composition and phenolic substrate (Lee *et al*., 2013). It has been shown that PBP formation from phenolic substrate can be independent of PPO activity and auto-catalytic; therefore, the total amount of enzyme may not be as important as the protein composition and the phenolic content (Kagan *et al*., 2016; Lee *et al*., 2013). In an experiment looking to protect C18 PUFA rich oils using protein extracts from vegetable sources Gadeyne *et al*. (2016) found that the effectiveness to protect emulsified lipid (PBP) against *in vitro* ruminal biohydrogenation largely depended on the protein concentration and type when adding phenolic substrate and was not related to PPO activity. It is therefore not surprising that in the current study no difference was found between RC+ and RC− due to the comparable protein content and although the phenolic contents differed they were low and may represent sampling loss (as already discussed) resulting in a comparable level of PBP protection despite differences in PPO enzyme content. This was further exemplified in the similar AA digestibilities between RC+ and RC− especially in relation to likely phenol binding sites (sulphur-containing AA) which indicates comparable protein complexing, despite different levels of PPO activity.

The proportions of C18:1 *cis* isomers in the duodenal digesta are similar to those previously reported when beef steers consumed either RC or PRG and can be explained by differences in C18:1 *cis* intake driven by C18:1n-9 (*cis*-9; Lee *et al*., 2006b and 2014). The differences in the proportions of C18:1 *trans* isomers between the RC silages and PRG in contrast could not be explained by intake differences, other than the higher level of *trans*-15 between RC− and RC+, which is likely an artefact due to the unresolved peak between *cis*-10 and *trans*-15. Similar C18:1 *trans* patterns have been reported previously in animals consuming grass or RC silage and may relate to changes in rumen microbial ecology (Lee *et al*., 2006b and 2014). Huws *et al*. (2010) demonstrated different rumen microbial communities developed when steers were offered either grass or RC silage, especially in relation to known lipolytic microbial communities. It is therefore possible that dietary changes, such as introducing RC, could also influence other, as yet uncultured, biohydrogenating microbial communities (Huws *et al*., 2011). Halmeimies-Beauchet-Filleau *et al*. (2013) also reported lower lipolytic activity across the rumen when RC compared to grass silages were fed in combination with concentrate (60 : 40; forage : concentrate, DM basis) which they attributed the increased PUFA flow on the RC diet. However, Halmeimies-Beauchet-Filleau *et al*. (2013) did not report the same shift in biohydrogenation intermediates, as in the current study, which may suggest a smaller impact on the microbial biohydrogenating community when concentrate is included in the ration. This altered rumen microbial community between grass and RC therefore may play a role in the observed elevated PUFA flow through lower microbial lipolytic activity in the rumen and requires further investigation.

## Conclusion

Small differences were observed between RC+ and RC− in rumen and whole tract N metabolism reflecting little advantage in selecting RC for high PPO activity to improve NUE. Red clover silage significantly reduced C18 PUFA biohydrogenation compared to PRG. However, no advantage was observed between RC silage with higher PPO activity in further reducing C18 PUFA biohydrogenation. The level of protein and C18 PUFA protection in the rumen is not related to PPO activity in the fresh crop and may more reflect a balance between phenolic substrate supply and enzyme activity driving protein complexing and/or changes in rumen microbial lipolytic communities, both mechanisms are discussed. However, total protein content is tantamount to influencing NUE of forage crops.

## Supplementary materials

To view supplementary material for this article, please visit https://doi.org/10.1017/S1751731118003294
